# Editorial: Prognostic biomarkers for oral cancer

**DOI:** 10.3389/froh.2022.994387

**Published:** 2022-09-07

**Authors:** Sabrina Wurzba, Tuula Anneli Salo, Ricardo D. Coletta

**Affiliations:** ^1^Lady Davis Institute for Medical Research and Segal Cancer Center, Jewish General Hospital, Montreal, QC, Canada; ^2^Department of Otolaryngology-Head and Neck Surgery, McGill University, Montreal, QC, Canada; ^3^Cancer and Translational Medicine Research Unit, Faculty of Medicine and Medical Research Centre Oulu, Oulu University Hospital, University of Oulu, Oulu, Finland; ^4^Department of Pathology, Institute of Oral and Maxillofacial Disease, Helsinki University Hospital, University of Helsinki and HUSLAB, Helsinki, Finland; ^5^Department of Oral Diagnosis and Graduate Program in Oral Biology, School of Dentistry, University of Campinas, São Paulo, Brazil

**Keywords:** prognosis, oral cancer, biomakers, therapeutic potential, tumor microenvironment

Cancers in the oral cavity, mainly represented by squamous cell carcinomas, are among highly prevalent tumors worldwide, representing the leading cause of cancer-mortality in certain regions in the world such as South and Southeast Asia. It is a major and emergent public health issue, once the late diagnosis, aggressive clinical behavior and the high rates of morbidity and mortality are main features of this disease. The main prognostic factor for oral cancer is encompassed by the classical TNM classification system: tumor size (T), regional nodal involvement (N) and the presence or absence of distant metastasis (M). Although the system is imperfect, partly because tumors with similar morphology and stage may behave differently due to their differing biological characteristics, it is widely used in treatment planning, prognostication, and comparison of outcomes. Many research efforts have been designed to identify reliable and unequivocal diagnostic and prognostic biomarkers to understand molecular and cellular mechanisms, which drive oral cancer initiation, maintenance and tumor progression. While the eight hallmarks of cancer helped to distill this complexity into an increasingly logical science, there are new parameter(s), such as the tumor microenvironment, to add an incredible value to that endeavor, and then to more fully understand mechanisms of cancer development, malignant tumor progression, and apply that knowledge to cancer medicine. In this special Research Topic, we presented four articles with the common goal of delivering information directly linked to the patients' outcomes, including tumor progression, cancer relapse and/or metastasis and therapeutic response. The findings confirmed that there is great value in studying biomarkers that could potentially be useful to increase the predictive impact of molecular and clinicopathological features to better forecast patients' outcomes.

The first published article by Wahab et al. investigated the prognostic impact of the astroprincin (APCN or also called FAM171A1), an astrocytic protein overexpressed in several tumors. The authors did not find a statistically significant association of the immunohistochemical expression of APCN and overall survival or disease free-survival in 76 oral cancer samples. They suggested that a large multicenter cohort study might be considered for future investigations to provide prognostic evaluation. Nowadays, there has been a significant progress in many molecular aspects involving protein expression and gene profiling, and investigations of tumor biomarkers are necessaries as they influence the prognosis of oral cancer and provide the potential to improve treatment through targeted therapy, but validation on well-designed large, prospective, multicenter clinical cohort-based studies is imperative.

The study conducted by Hano et al. revelated that lymph node metastasis is closely associated with the immunohistochemical levels of two endoplasmic reticulum (ER) stress-related proteins (Clptm1L and TMEM207) in 89 patients with oral squamous cell carcinoma from multiple locations. The authors indicated that the double positive Clptm1L-TMEM207 immunoreactivity had significant prognostic value in patients with oral cancer. It is interesting to highlight that most single biomarkers studies have not reached the level of cancer specificity and sensitivity required for routine clinical use in early detection and screening purposes. So, a growing confluence of scientific data and results point to combinations of biomarkers to provide more superior results than single markers alone.

Ndayisabye et al. conducted a cross-sectional research design on 311 medical records from patients from Rwanda Military Hospital in order to determine the clinical factors associated with poor prognosis. They showed that the outcome and the high rates of mortality are associated with religion and educational level of the patients as well as the location of the living province. Nevertheless, common clinical factors, such as age, gender, marital status, primary site, and wealth index were not significant in this population. They called for the attention of policy makers, scientists, and clinicians from public and private organizations to take into account these identified parameters to promote more clinical research in oral cancer in Sub-Saharan countries for earning knowledge, and then make significant contributions to improve information, education about oral cancer prevention and early diagnosis.

Duhen et al. discussed how the immune environment of tumors develops, the critical immune cell populations in advanced cancers, and how immune interventions can manipulate the immune environment of patients with potentially malignant lesions or advanced oral cancers to improve the therapeutic outcomes. Accumulating evidence point toward a complex and dynamic cross-talk between immune system and tumor cells, which may be responsible for regulating tumor growth and metastasis. Increased understanding of the biochemical nature of tumor antigens and the molecular mechanisms responsible for innate and adaptive immune cell activation has revolutionized the fields of tumor immunology and immunotherapy. Indeed, immunotherapy is rapidly altering the therapeutic landscape in head and neck cancer. Consequently, through a deeper understanding of the complex relationship between the immune system and tumor cells could inform the delivery of effective immunotherapeutic approaches to improve overall survival and patient's quality of life.

It is important to note that despite advances in oral science and treatment approaches, the rates of both overall survival and disease-free-survival of oral cancer patients are poor and remain stagnant at 40–50% in the last four decades. In this sense, early diagnosis still offers the best chance of cure. There are a lot of innovative studies taking place at outstanding universities and research institutes. The characterization of biomarkers with prognostic impact offers unprecedented prospects for translational research, with direct impact on therapeutic decision-making, post-therapeutic monitoring, and potential development of novel therapeutic targets. We have only begun to scratch the surface of this field in oral cancer and are poised for more discoveries as whole-transcriptome sequencing becomes more commonplace in the clinic and more data is available. Congratulations to the research partners (Wahab et al.; Hano etal.; Ndayisabye et al.; Duhen et al.), who shared their discoveries within this topic. We believe this research area is one of the top priorities in oral cancer to revert the obscure scenario of poor outcomes. The most recent perspective and progress in biomarkers research for oral cancer will continue to be explored and shared in special Research Topics by the most influential clinical and basic research teams. The development of a personalized medicine in oral health is directly linked with the discovery of robust biomarkers and new therapeutic targets.

## Author contributions

All authors listed have made a substantial, direct, and intellectual contribution to the work and approved it for publication.

## Conflict of interest

The authors declare that the research was conducted in the absence of any commercial or financial relationships that could be construed as a potential conflict of interest.

## Publisher's note

All claims expressed in this article are solely those of the authors and do not necessarily represent those of their affiliated organizations, or those of the publisher, the editors and the reviewers. Any product that may be evaluated in this article, or claim that may be made by its manufacturer, is not guaranteed or endorsed by the publisher.

